# Polymorphisms of microRNA Sequences or Binding Sites and Lung Cancer: A Meta-Analysis and Systematic Review

**DOI:** 10.1371/journal.pone.0061008

**Published:** 2013-04-16

**Authors:** Zhiwei Chen, Ling Xu, Xiangyun Ye, Shengping Shen, Ziming Li, Xiaomin Niu, Shun Lu

**Affiliations:** 1 Shanghai Lung Tumor Clinical Medical Center, Shanghai Chest Hospital, Shanghai Jiao Tong University, Shanghai, China; 2 Department of Oncology, Longhua Hospital, Shanghai University of Traditional Chinese Medicine, Shanghai, China; University of Porto, Portugal

## Abstract

**Objective:**

Functional single nucleotide polymorphisms (SNPs) of microRNA (miRNA) sequences or binding sites (miRNA-SNPs) are associated with lung cancer risk and survival. The objective of this study was to systematically review genetic association studies about miRNA-SNPs in lung cancer.

**Methods:**

Eligible genetic association studies were retrieved from databases of PubMed, EMBASE, China National Knowledge Infrastructure and SinoMed. Two investigators selected related studies and assessed methodological quality independently. Quantitative data synthesis was conducted for common SNPs of miRNA (miRNA-196a2 rs11614913, miRNA146a rs2910164, miRNA149 rs2292832, miRNA-605 rs2043556 and miRNA499 rs3746444). GRADE profiler was used to grade the quality of evidence for each miRNA-SNP.

**Results:**

15 eligible studies and 27 miRNA-SNPs were retrieved and 10 miRNA-SNPs were reported with a significant association with susceptibility to or survival of lung cancer. Methodological quality of eligible studies was adequate with an average score of 8.5. miRNA-196a2 rs11614913 polymorphism was associated with increased lung cancer risk (homozygote comparison, OR = 1.299, 95% CI: 1.096–1.540; dominant model, OR = 1.217, 95% CI: 1.041–1.421) and decreased survival. And according to GRADE profiler, quality of evidence was moderate for MYCL1 rs3134615, while quality of the other significant associations was low.

**Conclusions:**

Based on this first systematic review about miRNA-SNPs in lung cancer, quality of evidence was low for most genetic association studies. Polymorphisms of miRNA-196a2 rs11614913 and MYCL1 rs3134615 could be potential biomarkers of lung cancer.

## Introduction

Lung cancer is the leading cause of cancer-related death worldwide [1]. It is widely accepted that both genetic susceptibility and environment exposure contribute to lung cancer and a lot of single nucleotide polymorphisms (SNPs) are associated with lung cancer risk [2], [3]. Additionally, recent studies suggested that functional SNPs occurring in micorRNA (miRNA) sequences or binding sites of miRNAs, namely the miRNA-SNPs, were associated with susceptibility to lung cancer [4]–[7], which highlighted a new paradigm for genetic susceptibility.

miRNAs, a kind of endogenous small non-coding RNAs, are about ∼22 nucleotides in length and function as negative regulators of post-transcriptional gene expression [8]–[10]. Mature miRNAs primarily target the 3′ untranslated region (3′UTR) of their target mRNA, leading to mRNA degradation or suppression of translation [8], [11]. miRNAs are crucial for regulation of various biological processes, such as gene regulation, tumorigenesis, proliferation, apoptosis, and metabolism [12]–[14]. It is estimated that at least 30% of protein coding genes are regulated by miRNAs [15] and on the other hand, a single miRNA can also bind to 3′UTR of many mRNA [16]. SNPs occurring in miRNA sequences can affect processing and binding ability of mature miRNAs. Functional SNPs of miRNA-146a [17], miRNA-499 [18], and miRNA-196a2 [19] have been found associated with cancer susceptibility, including lung cancer. Several studies [4]–[6] have investigated the correlation between rs11614913 miRNA-196a2 polymorphism and lung cancer risk; however, the results are inclusive.

In addition to polymorphisms of miRNAs, polymorphisms in the binding sites of miRNAs can also contribute to susceptibility of lung cancer. Chin and colleagues [20] identified a novel SNP in the 3′UTR of KRAS gene which altered the binding affinity of miRNA let-7. They also found that this SNP (LCS6) was associated with lung cancer risk in low-dose smokers. Furthermore, functional miRNA-SNPs may be potential biomarkers to predict clinical outcome of lung cancer.

The current review is the first systematic assessment about SNPs of miRNA sequences and binding sites in lung cancer. In this study, the primary objective was to evaluate the association strength of common miRNA-SNPs with susceptibility to lung cancer. And the secondary objective was systematically reviewing current studies about miRNA-SNPs and risk or clinical outcome of lung cancer and assessing the level of evidence using GRADE profiler.

## Methods

### Searching Strategy

Eligible studies were extracted by searching electronic databases. A comprehensive search of major databases was conducted, i.e. PubMed, EMBASE, China National Knowledge Infrastructure (CNKI) and SinoMed (CBM) were searched. The following key words and medical subheadings were used, “MicroRNAs”, “lung neoplasms”, and “single nucleotide polymorphism”. Alternative spellings were also considered. The last search was performed on December 3, 2012, and there was no limit of languages.

### Inclusion and Exclusion Criteria

Eligible studies were selected by two reviewers (Chen and Xu) independently according to following inclusion criteria: 1) investigating miRNA-SNPs and lung cancer risk or clinical outcome; 2) published full-text articles. After the primary screening of titles and abstracts, full-text articles were retrieved and further reviewed for eligibility. The two reviewer reached consensus on each study.

### Data Extraction

Two reviewers (Chen and Xu) extracted data from eligible studies in duplicate with a standard data-collection form, and reached consensus on each item. The following data was extracted: name of first author, year of publication, country, ethnicity, number of participants, comparison model, and odds ratios (OR) or hazard ratios (HR) with 95% confidence intervals (CI). For the studies investigating SNPs of miRNA sequences and lung cancer risk, detailed genotype was also collected. Ethnicity descents were simply classified as European and Asian. In the study reported by Nelson and colleagues [21], no HR or 95% CIs were available, thus the HR and 95% CIs were estimated from Kaplan-Meier curves using the method proposed by Tierney et al [22].

### Methodological Quality Assessment

Two methodological quality scales were adopted to assess quality of eligible studies. For studies about miRNA-SNPs and lung cancer risk, a quality scale (“Table S2: Methodological quality assessment scale for risk”) reported by previous meta-analysis [19] was used. For studies concerning survival of lung cancer and miRNA-SNPs, another quality scale (modified from previous study [23], “Table S3: Methodological quality assessment scale for survival”) was applied. Quality scores of both scales range from 0 to 10, and a high score indicates good quality. Methodological quality of eligible studies was assessed by two investigators independently (Shen and Ye).

### GRADE Quality Assessment

GRADE profiler (version 3.6) was adopted to grade quality of evidence for each association. Factors those would upgrade (large effect, plausible confounding, and dose response) and downgrade (risk of bias, inconsistency, indirectness, imprecision, and publication bias) quality of evidence was evaluated. Two investigators (Shen and Ye) assessed quality independently and solved disagreement by discussion.

### Statistical Analysis

ORs and 95% CIs were calculated to estimate the association strength of common miRNA-SNPs with lung cancer risk. For lung cancer risk, data synthesis was performed for 5 common SNPs of miRNA (miRNA-196a2 rs11614913, miRNA146a rs2910164, miRNA149 rs2292832, miRNA-605 rs2043556 and miRNA499 rs3746444) but quantitative synthesis was not performed for prognosis with limited data. A number of 4 comparison models were conducted for each SNP, namely homozygote comparison (M1: AA vs. aa), heterozygote comparison (M2: Aa vs. aa), dominant model (M3: AA/Aa vs. aa), and recessive model (M4: AA vs. Aa/aa) (A: variant allele, a: wild allele). Random-effects model (DerSimonian-Laird method) was adopted in each comparison to minimize bias, concerning the limited number of studies. A 95% CI without 1for OR indicated a significant association with lung cancer risk. Heterogeneity between studies was tested by chi-square based Q test and a P<0.1 indicated significant heterogeneity [24]. Given limited number of studies included in data synthesis, subgroup analysis and sensitivity analysis was not performed. Publication bias was tested by Begg’s test and the Egger’ test and a P value less than 0.05 was considered significant [25].

All P values were two-side. All statistical analyses were calculated with STATA software (version 10.0; StataCorp, College Station, Texas USA).

## Results

### Selection of Eligible Studies

**Figure 1:**
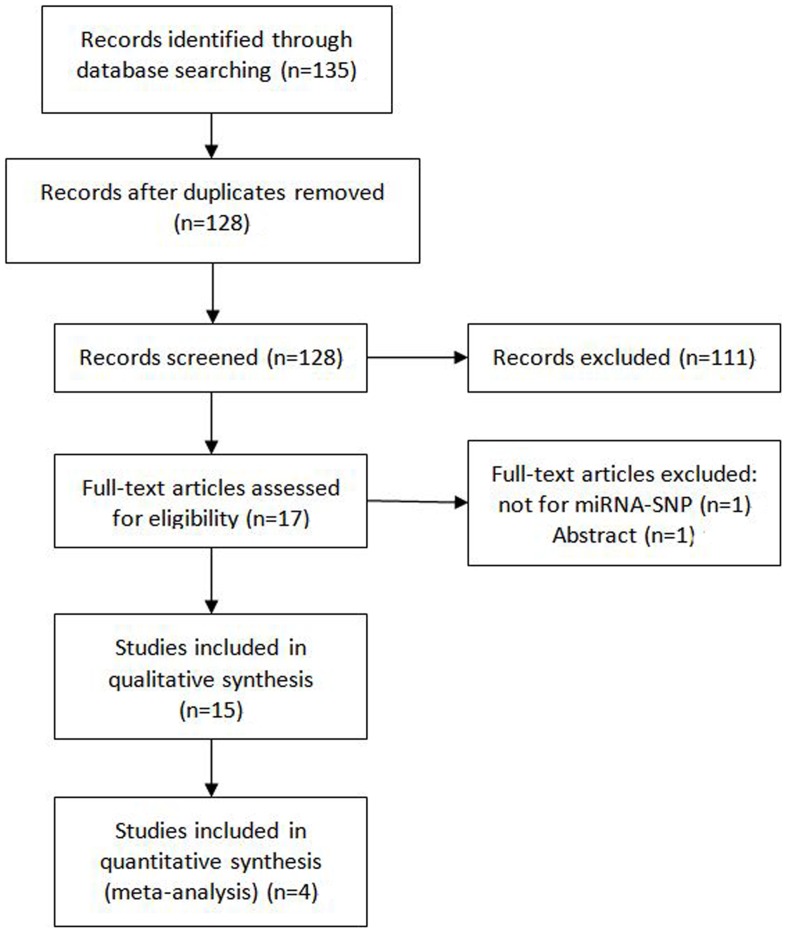
Flow chart.

A total of 135 records were retrieved from databases. After primary screening of titles and abstracts, 17 full-text articles were further reviewed for eligibility. Two articles were excluded for the reason of being abstract [26] and not about miRNA-SNPs [27]. Thus, 15 eligible studies [4]–[7], [20], [21], [28]–[36] were retrieved and reviewed and 4 of them [4]–[7] were included in quantitative synthesis (shown in Figure 1).

### Characteristics of Eligible Studies

Table 1 shows characteristics of eligible studies. As shown, 12 of 15 studies were conducted in Asian population and only 3 studies [20], [21], [29] were performed in European population, which may lead to a discrepancy of ethnicity. Sample sizes of included studies were adequate except for one study reported by Ding et al [30]. All studies were of high methodological quality with an average quality score of 8.5.

**Table 1:** Characteristics of eligible studies.

Author	SNP	Year	Country	Ethnicity	Participants	Quality Score
Zhan X	miRNA-196a2 rs11614913	2012	China	Asian	442	10
Ding C	rs16917496, 3′UTR of SET8	2012	China	Asian	44	6
Yoon KA	miRNA-219-1 rs213210, miRNA-27a rs895819, miRNA-492 rs2289030, miRNA-146a rs2910164, miRNA-423 rs6505162, miRNA-26a-1 rs7372209	2012	Korea	Asian	388	8.5
Zhang S	rs465646, 3′UTR of REV3L	2012	China	Asian	2136	10
Yang L	rs2735383, 3′UTRof NBS1	2011	China	Asian	3238	9
Campayo M	rs36603′UTR of KRT81	2011	Spain	European	175	8.5
Xiong F	rs3134615, 3′UTR of MYCL1	2011	China	Asian	1424	8.5
Hu Z	let-7a-2 rs629367,miRNA-1–2 rs9989532, miRNA-29c rs2724377,miRNA-30c-1rs928508,miRNA-31rs13283671,miRNA-33rs9620000, miRNA-125brs2241490, miRNA-145 rs353291, miRNA-193brs30236, miRNA-302dand miRNA-367 rs13136737, and miRNA-378 rs1076064	2011	China	Asian	923	9.5
Zhang MW	miRNA-605 rs2043556, miRNA-149 rs2292832	2011	China	Asian	487	8.5
Hong YS	miRNA-196a2 rs11614913	2011	Korea	Asian	834	7
Kim MJ	miRNA-196a2 rs11614913	2010	Korea	Asian	1294	8
Nelson HH	3′UTR of KRAS(LCS6)	2009	USA	European	218	7
Tian T	miRNA-146a rs2910164, miRNA-149 rs2292832, miRNA-196a2rs11614913, miRNA-499 rs3746444	2009	China	Asian	2093	9.5
Chin LJ	3′UTR of KRAS(LCS6)	2008	USA	European	4245	9
Hu Z	miRNA-146a rs2910164, miRNA-149 rs2292832, miRNA-196a2rs11614913, miRNA-499 rs3746444	2008	China	Asian	931	8.5

### Association Strength of miRNA-SNPs and Lung Cancer

A total of 27 miRNA-SNPs were reported in current studies and 10 of them had a significant association with lung cancer risk or survival.

**Figure 2:**
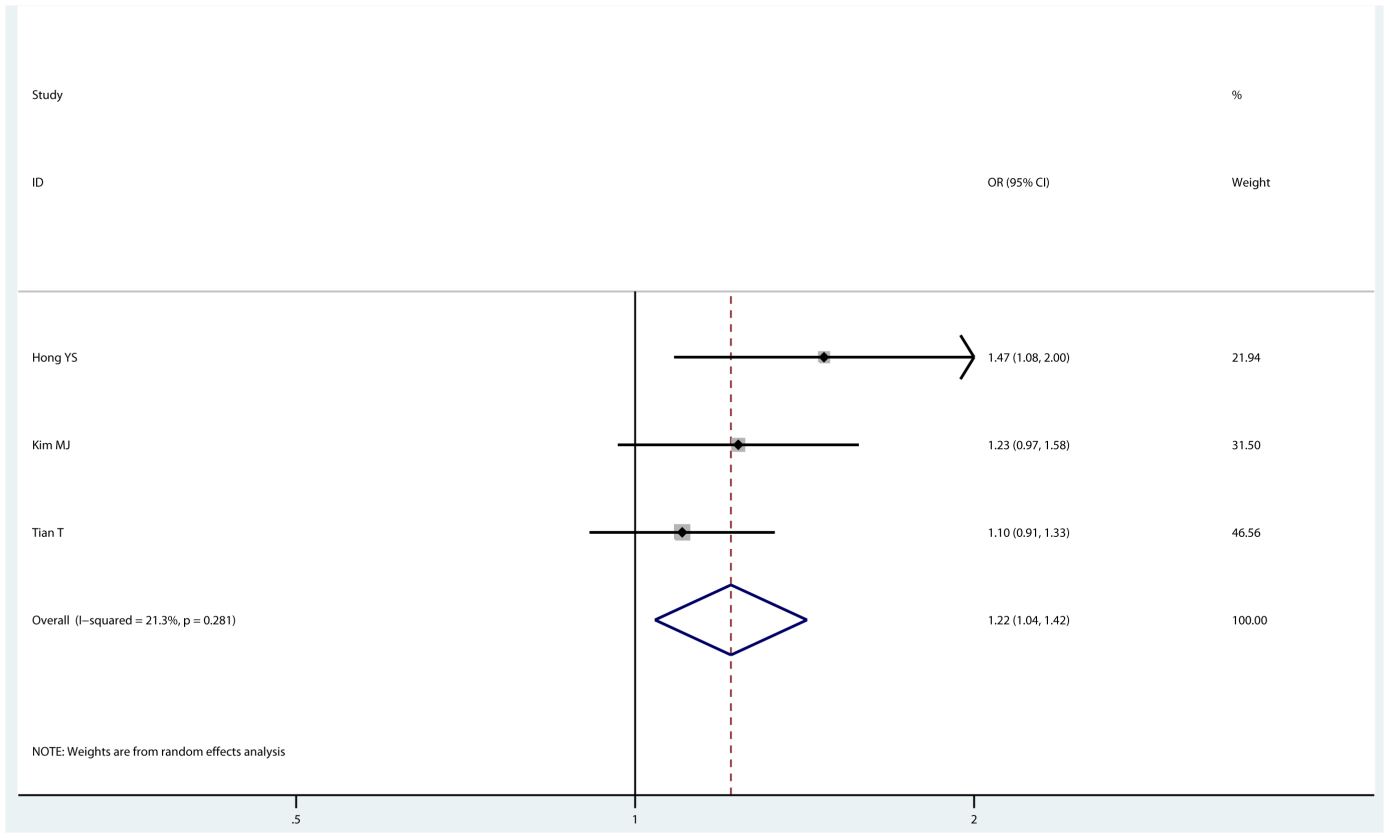
Forrest plot of miRNA-196a2 rs11614913 (dominant model). Estimated by random-effect model (CC+CT vs. TT).

Five common SNPs occurred in miRNA sequences were included in quantitative synthesis, and detail results were shown in Table 2. miRNA-196a2 rs11614913 polymorphism has been demonstrated with increased risk of many cancers, but only 3 studies [4]–[6] investigated rs11614913 polymorphism and lung cancer risk. By pooling eligible data, we found that the variant C allele increased susceptibility to lung cancer in homozygote comparison (OR = 1.299, 95% CI: 1.096–1.540, P_heterogeneity_ = 0.895) and dominant model (OR = 1.217, 95% CI: 1.041–1.421, P_heterogeneity_ = 0.281, P_Begg_ = 0.296, P_Egger_ = 0.094; Figure 2). However, according to GRADE profiler, the quality of this association was low. No significant association with lung cancer risk was detected for miRNA-149 rs2292832 (dominant model, OR = 1.068, 95% CI: 0.913–1.248, P_heterogeneity_ = 0.975), miRNA-146a rs2910164 (dominant model, OR = 1.052, 95% CI: 0.878–1.259), miRNA-499 rs3746444 (dominant model, OR = 0.956, 95% CI: 0.788–1.161), or miRNA-605 rs2043556 (dominant model, OR = 0.840, 95% CI: 0.587–1.203). No evidence of publication bias was detected.

**Table 2:** Meta-analysis results of common SNPs of miRNAs.

miRNA	M1	M2	M3	M4	GRADE
miRNA-146a rs2910164	1.125(0.873,1.450)	1.027(0.849,1.243)	1.052(0.878,1.259)	1.107(0.881,1.391)	very low
miRNA-149 rs2292832	1.159(0.891,1.508)	1.047(0.889,1.234)	1.068(0.913,1.248)	1.131(0.881,1.452)	very low
miRNA-196a2 rs11614913	1.299(1.096,1.540)*	1.204(0.956,1.516)	1.217(1.041,1.421)*	1.171(0.996,1.378)	low
miRNA-499 rs3746444	0.892(0.508,1.568)	0.963(0.788,1.177)	0.956(0.788,1.161)	0.901(0.514,1.579)	very low
miRNA-605 rs2043556	0.708(0.337,1.484)	0.864(0.595,1.256)	0.840(0.587,1.203)	0.754(0.366,1.553)	very low

Results are shown as OR 95% CI; M1: homozygote comparison; M2 heterozygote comparison; M3: dominant model; M4: recessive model;

* for significant difference.

Hu and colleagues [31] investigated the correlation between common SNPs of miRNAs (miRNA-196a2 rs11614913, miRNA-149 rs2292832, miRNA-146a rs2910164, and miRNA-499 rs3746444) and long-term survival of non-small cell lung cancer (NSCLC). They found rs2910164 and rs11614913 were associated with survival and the quality of this association was low according to GRADE pofiler (Table 3). But the predictive role of miRNA-196a2 rs11614913 polymorphism differed among NSCLC and small cell lung cancer (SCLC). Zhan and colleagues [28] evaluated the predictive value of rs11614913 polymorphism in SCLC. Zhan [28] did not observe a significant association of rs11614913 polymorphism with response to chemotherapy in SCLC (OR = 0.88, 95% CI: 0.50–1.55); however, rs11614913 polymorphism could predict overall toxicity (OR = 1.73, 95% CI: 1.10–2.71).

**Table 3:** miRNA-SNPs and lung cancer.

Author	SNP	Correlation	Comparison Model and P value	GRADE quality	Reason
Zhan X	miRNA-196a2 rs11614913	response to chemotherapy	CC vs. CT/TT; OR, 0.88, 95% CI:0.50–1.55	very low	imprecision
		overall toxicity	CC vs. CT/TT; OR, 1.73, 95% CI:1.10–2.71*	low	
Yoon KA	miRNA-219-1 rs213210	recurrence-free survival	AG/GG vs. AA; HR,1.17; 95% CI:0.67–2.03	very low	imprecision
	miRNA-27a rs895819		CT/CC vs. TT; HR,0.98; 95% CI: 0.61–1.60	very low	imprecision
	miRNA-492 rs2289030		CG/CC vs. GG; HR,1.04; 95% CI:0.65–1.69	very low	imprecision
	miRNA-146a rs2910164		CG/GG vs. CC; HR,0.52; 95% CI:0.32–0.85*	low	
	miRNA-423 rs6505162		AC/AA vs. CC; HR,1.09; 95% CI:0.67–1.77	very low	imprecision
	miRNA-26a-1 rs7372209		CT/TT vs. CC; HR,1.16; 95% CI: 0.72–1.86	very low	imprecision
Hu Z^c^	miRNA-30c-1 rs928508	survival	AG/GG vs. AA; HR,0.73; 95% CI:0.61–0.89*	low	
	let-7a-2 rs629367		AC/CC vs. AA; HR,0.91; 95% CI:0.66–1.26	very low	imprecision
	miRNA-1–2 rs9989532		AG/GG vs. AA; HR,0.78; 95% CI:0.49–1.24	very low	imprecision
	miRNA-29c rs2724377		GG vs. AA/AG; HR,1.35; 95% CI:0.49–3.68	very low	imprecision
	miRNA-31 rs13283671		GG vs. AA/AG; HR,1.82; 95% CI:1.05–3.18* ^a^	very low	
	miRNA-33 rs9620000		AG vs. AA; HR,1.23; 95% CI:0.83–1.82	very low	imprecision
	miRNA-125b rs2241490		AA vs. GG/GA; HR,1.20; 95% CI:0.72–2.01	very low	imprecision
	miRNA-145 rs353291		AG/GG vs. AA; HR,1.47; 95% CI:1.04–2.07* ^a^		
	miRNA-193b rs30236		AA vs. GG/GA; HR,1.02; 95% CI:0.63–1.68	very low	imprecision
	miRNA-378 rs1076064		AG/GG vs. AA; HR,1.10; 95% CI:0.77–1.58	very low	imprecision
	miRNA-302d and miRNA-367 rs13136737		CA/AA vs. CC; HR,0.92; 95% CI:0.67–1.25	very low	imprecision
Hu Z	miRNA-146a rs2910164	survival	GG vs. CC; HR,1.28; 95% CI:0.89–1.82	very low	imprecision
	miRNA-149 rs2292832		TT vs. GG/GT; HR,1.29; 95% CI:1.01–1.65*	low	
	miRNA-196a2 rs11614913		CC vs. TT/CT; HR,1.76, 95% CI:1.34–2.33*	low	
	miRNA-499 rs3746444		GG vs. AA; HR,1.24, 95% CI:0.55–2.83	very low	imprecision
Ding C	rs16917496, 3′UTR of SET8	2 year survival	CT/CC vs. TT; HR,0.59, 95% CI:0.33–1.06* ^b^	very low	imprecision
Zhang S	rs465646, 3′UTR of REV3L	susceptibility	TC/CC vs. TT; OR,0.71; 95% CI:0.59–0.85*	low	
Yang L	rs2735383, 3′UTRof NBS1	susceptibility	CC vs. GG/GC; OR,1.40; 95% CI:1.18–1.66*	low	
Campayo M	rs36603′UTR of KRT81	time to recurrence	CG/GG vs. CC; HR,0.51; 95% CI:0.35–0.74* ^b^	low	
Xiong F	rs3134615, 3′UTR of MYCL1	susceptibility	GT/TT vs. GG; OR,2.08; 95% CI:1.39–3.12*	moderate	large effect
Nelson HH	3′UTR of KRAS(LCS6)	5 year survival	TG/GG vs. TT; HR,0.99; 95% CI:0.56–1.74^b^		imprecision
Chin LJ^d^	3′UTR of KRAS(LCS6)	susceptibility	TG/GG vs. TT; OR,1.36; 95% CI:1.07–1.73*	low	

* significant association;

a: different results between screening set and validation set;

b: HR and 95% CI estimated form Kaplan-Meier curve;

c: data from the validation set; d: data from low-dose smokers of Boston case-control study.

Other SNPs of miRNAs were also investigated. Hu et al [32] identified a novel SNPs of miRNA (miR-30c-1 rs928508) in Chinese population, which was associated with survival in NSCLC. Yoon et al [35] explored the prognostic impact of several miRNA polymorphisms in resected NSCLC, and only miR-146a rs2910164 was of predictive value. These two studies were carried out in Asia population and the quality of evidence was low.

Several SNPs of miRNA binding sites were studied and significant association with susceptibility and survival was identified. A total of 6 SNPs in the miRNA binding sites were found associated with lung cancer risk or survival so far (Table 3) but only the association of rs3134615 (3′UTR of MYCL1; OR = 2.08; 95% CI: 1.39–3.12) had a moderate quality [33] (Table 3). Notably, genes in which the polymorphisms of binding sites occurred are oncogenes or involved in DNA repair pathways. For example, REV3L is responsible for DNA repairing [36] and MYCL1 is a member of MYC oncogenes family [33]. Additionally, these polymorphisms could affect gene functions and protein expression, which was in consistent with predictive role of these miRNA-SNPs.

Chin et al [20] identified a polymorphism in the 3′UTR of KRAS (LCS6) and the LCS6 polymorphism increased lung cancer risk in low-dose smokers (GRADE quality of evidence, low) but this association was not significant in overall population (GRADE quality of evidence, very low). Thereafter, Nelson and colleagues [21] investigated LCS6 polymorphism and survival of lung cancer and failed to find any significant association (HR = 0.99, 95% CI: 0.56–1.74). Intriguingly, these two studies about LCS6 polymorphism were both carried out in Europe population, and the LCS6 polymorphism has not been validated in other ethnicities.

Each of the 6 SNPs in miRNA binding sites was reported in one single study, and due to imprecision, these studies could not provide evidence of high quality.

## Discussion

In this first extensive systematic review about miRNA-SNPs in lung cancer, we identified 15 genetic association studies on this topic and evaluate the quality of evidence of 27 miRNA-SNPs. Methodological quality of eligible studies was adequate and GRADE profiler suggested that the association level of most miRNA-SNPs was low, except for rs3134615 polymorphism in the 3′UTR of MYCL1 [33] (moderate).

Numerous studies have investigated the common SNPs of miRNA and risks of different kinds of cancer. In this study, we found polymorphism of miRNA-196a2 increased lung cancer risk in homozygote comparison (OR = 1.299, 95% CI: 1.096–1.540) and dominant model (OR = 1.217, 95% CI: 1.041–1.421). And the polymorphisms of miRNA-146a, miRAN-149, and miRNA-499 did not show any significant association, though altered risk was observed in other cancers. However, in the study by Hu et al [31], polymorphisms of miRNA-149 and miRNA-196a2 were significantly correlated with long-term survival of NSCLC. Additionally, Zhan and colleagues [28] showed that miRNA-196a2 rs11614913 could predict overall toxicity of chemotherapy in NSCLC. These results suggest that miRNA-196a2 rs11614913 polymorphisms may be a potential biomarker for risk of lung cancer and clinical outcome of NSCLC.

Six SNPs in the binding sites of miRNA were retrieved from eligible studies and all of them were associated with susceptibility to or survival of lung cancer. But only rs3134615 (3′UTR of MYCL1) showed moderate quality [33], which highlights the role of rs3134615 as a potential biomarker in SCLC. As few studies investigated genetic variation in SCLC, the polymorphism of MYCL1should further validated in different ethnicities with a large sample size. Ding and colleagues indentified a SNP at rs16917496, the 3′UTR of SET8 [30], and found it associated with survival of SCLC. Intriguingly, SET8, a methyltransferase, modulates p53 expression [37]. Additionally, the prognostic value of KRT81 rs3660 polymorphism was more marked in squamous cell cancer [29]. Although the association level of KRT81 rs3660 and SET8 rs16917496 was low according to GRADE profiler, further studies are warrant.

These SNPs of miRNA binding sites were located in oncogenes or critical genes involved in DNA repairing pathways. These genes are critical for carcinogenesis and integrity of genome. Based on features of these host genes, we might infer that other SNPs in the 3′UTR of oncogenes or DNA repair genes would be possibly of predictive value for cancer risk or survival.

Based on our systematic review, some weakness of current studies was identified. At first the association level identified by current studies was low because of imprecision according to GRADE profiler. Additionally, most SNPs were identified by one single study and no further validation was available. Secondly, most studies were carried out in Asian population and only 3 studies [20], [21], [29] and 2 SNPs were performed in European polulation. Furthermore, of 7 studies [20], [21], [29], [30], [33], [34], [36] about SNPs of miRNA binding sites, only one study [33] reported insignificant correlation with lung cancer survival, which indicated the existence of publication bias.

In conclusion, we identified 10 SNPs of miRNA sequences or binding sites that were associated with lung cancer risk or survival. However, the quality of evidence was low according to GRADE profiler. Weakness of current studies was stressed and translational potential of miRNA-196a2 rs11614913 and MYCL1rs3134615 was also discussed.

## Supporting Information

Table S1 **PRISMA checklist.**
(DOC)

Table S2 **Methodological quality assessment scale for risk.**
(DOC)

Table S3 **Methodological quality assessment scale for survival.**
(DOC)
