# Supplementary material for: Polymorphisms of microRNA Sequences or Binding Sites and Lung Cancer: A Meta-Analysis and Systematic Review
Source: PLoS One. 2013 Apr 16;8(4):e61008. doi: 10.1371/journal.pone.0061008 (PMC3628762; doi:10.1371/journal.pone.0061008)
Supplement: Table S3 — Methodological quality assessment scale for survival. (DOC) [file pone.0061008.s003.doc]

Table S3. Scale for methodological quality assessment of studies about cancer prognosis

| Criteria | Score |
| --- | --- |
| 1. Evaluation criteria |  |
| WHO/RECIST | 2 |
| Not described | 1 |
| 2. Chemotherapy regimens |  |
| Detailed | 1 |
| Not described | 0.5 |
| 3. Stage |  |
| Detailed | 1 |
| Not described | 0.5 |
| 4. Survival |  |
| Original data | 2 |
| Estimation from the Kapla-Meier curves | 1 |
| 5. Genotyping Methods |  |
| Illumina assays/MassArray | 2 |
| TaqMan | 2 |
| PCR-RFLP/LDR-PCR | 1 |
| 6. Sample Size |  |
| ≥150 | 2 |
| >100 and <150 | 1.5 |
| ≤100 | 1 |
